# ^64^Cu-ATSM and ^18^FDG PET uptake and ^64^Cu-ATSM autoradiography in spontaneous canine tumors: comparison with pimonidazole hypoxia immunohistochemistry

**DOI:** 10.1186/1748-717X-7-89

**Published:** 2012-06-15

**Authors:** Anders E Hansen, Annemarie T Kristensen, Jesper T Jørgensen, Fintan J McEvoy, Morten Busk, Albert J van der Kogel, Johan Bussink, Svend A Engelholm, Andreas Kjær

**Affiliations:** 1 Department of Radiation Oncology, Copenhagen University Hospital, Blegdamsvej 9, DK-2100, Copenhagen, Denmark; 2 Department of Small Animal Clinical Sciences, University of Copenhagen, Dyrlaegevej 16, DK-1870, Frederiksberg C, Denmark; 3 Cluster of Molecular Imaging, Faculty of Health and Medical Sciences, Blegdamsvej 3B, DK-2200, Copenhagen N, Denmark; 4 PET and Cyclotron Unit, Copenhagen University Hospital, Blegdamsvej 9, DK-2100, Copenhagen, Denmark; 5 Department of Experimental Clinical Oncology, Aarhus University Hospital, Norregade 44, DK-800, Aarhus, Denmark; 6 Department of Radiation Oncology, Radboud University Nijmegen Medical Centre, 874 Radboud, 9101, 6500 HB, Nijmegen, Netherlands

## Abstract

**Background:**

The aim of this study was to compare ^64^Cu-diacetyl-bis(*N*^4^-methylsemicarbazone) (^64^Cu-ATSM) and ^18^FDG PET uptake characteristics and ^64^Cu-ATSM autoradiography to pimonidazole immunohistochemistry in spontaneous canine sarcomas and carcinomas.

**Methods:**

Biopsies were collected from individual tumors between approximately 3 and 25 hours after the intravenous injection of ^64^Cu-ATSM and pimonidazole. ^64^Cu-ATSM autoradiography and pimonidazole immunostaining was performed on sectioned biopsies. Acquired ^64^Cu-ATSM autoradiography and pimonidazole images were rescaled, aligned and their distribution patterns compared. ^64^Cu-ATSM and ^18^FDG PET/CT scans were performed in a concurrent study and uptake characteristics were obtained for tumors where available.

**Results:**

Maximum pimonidazole pixel value and mean pimonidazole labeled fraction was found to be strongly correlated to ^18^FDG PET uptake levels, whereas more varying results were obtained for the comparison to ^64^Cu-ATSM. In the case of the latter, uptake at scans performed 3 h post injection (pi) generally showed strong positive correlated to pimonidazole uptake.

Comparison of distribution patterns of pimonidazole immunohistochemistry and ^64^Cu-ATSM autoradiography yielded varying results. Significant positive correlations were mainly found in sections displaying a heterogeneous distribution of tracers.

**Conclusions:**

Tumors with high levels of pimonidazole staining generally displayed high uptake of ^18^FDG and ^64^Cu-ATSM (3 h pi.). Similar regional distribution of ^64^Cu-ATSM and pimonidazole was observed in most heterogeneous tumor regions. However, tumor and hypoxia level dependent differences may exist with regard to the hypoxia specificity of ^64^Cu-ATSM in canine tumors.

## Background

The presence of hypoxic regions within solid tumors was recognized more than 50 years ago [1]. Hypoxic cancer cells are inherently resistant to treatment and the hostile microenvironment associated with them exerts a selective pressure that causes proteomic and genetic changes, which increase tumor aggressiveness [2,3].

Tumor hypoxia effects therapeutic outcome so diagnostic modalities that can identify hypoxia will benefit patients by stratifying for hypoxia adapted treatment. Several methodologies for the assessment of tumor hypoxia have been investigated, including positron emission tomography (PET). Hypoxia specific radiotracers, mostly 2-nitroimidazole derivates, such as ^18^ F-Fluoromisonidazole (F-MISO) are the most intensely studied [4-6]. Despite the generally positive correlation between the uptake of 2-nitroimidazoles and that of other hypoxic markers, their kinetics, slow hypoxia-specific retention and slow clearance of non-bound contaminating tracer are problematic [6]. Cu-diacetyl-bis(*N*^4^-methylsemicarbazone) (Cu-ATSM) is among the most promising alternatives to 2-nitroimidazole for hypoxia PET imaging. Cu-ATSM is a highly membrane permeable complex with a rapid blood clearance and high intertissue and intratumoral contrast. The proposed trapping mechanism of Cu-ATSM is indirectly linked to hypoxia, via chemical reduction from a cell membrane permeable to a non-permeable state. The exact mechanism by which Cu-ATSM is trapped in hypoxic cells or the oxygen-level required for accumulation is however not completely understood [7,8].

Invasive and non-invasive preclinical studies of Cu-ATSM have been conducted in xenografted animal models using various cancer lines. Cu-ATSM accumulation was positively correlated to hypoxia as measured by polarographic microelectrodes in a 9 L gliosarcoma rat model [9]. Additional studies have examined the uptake and distribution patterns of Cu-ATSM, to various non-invasive and invasive surrogate markers for hypoxia. Conflicting results have been obtained in these studies, which have questioned the universal hypoxia selectivity of the tracer and pointed towards potential tumor type dependent differences in accumulation kinetics [10-13]. These pre-clinical studies have underlined the need for additional invasive studies, preferably in a large spontaneous tumor model, to further investigate the distribution and uptake kinetics of Cu-ATSM and thus its usefulness as a non-invasive hypoxia radiotracer.

Pimonidazole belongs to the group of 2-nitroimidazoles and is a cell permeable hypoxia marker that is reduced and irreversibly trapped under hypoxic conditions (pO_2_ < 10 mmHg). Specific antibodies have been developed that targets pimonidazole adducts formed by nitroreductases under hypoxic conditions. Immunohistochemical analysis of pimonidazole adduct formations is therefore possible following systemic administration of pimonidazole [14,15].

The objective of this study was to compare uptake characteristics of pimonidazole immunohistochemistry to ^64^Cu-ATSM autoradiography and to PET uptake levels of ^64^Cu-ATSM and 2-deoxy-2-^18^ F]fluoro-D-glucose (^18^FDG) in spontaneous canine sarcomas and carcinomas. This study supplements previously reported non-invasive findings using Cu-ATSM and FDG as PET tracers in spontaneous canine tumors [16].

## Methods

### Study population

Spontaneous canine cancer patients with histologically diagnosed soft tissue sarcomas or carcinomas were eligible for inclusion. Owners of the dogs concerned provided written informed consent prior to inclusion in the study. Eight dogs met the inclusion criteria of this study (Table 1), dogs 1 to 6 also participated in a concurrent study [16]. The study protocol was approved by the ethical and administrative research committee at the Department of Small Animal Clinical Sciences, Faculty of Life Sciences, University of Copenhagen.

**Table 1:** Tumor characteristics

**Dog no.**	**Tumor type**	**Volume**	^**64**^**Cu-ATSM T**_**max**_**/M**_**mean**_	^**64**^**Cu-ATSM T**_**mean**_**/M**_**mean**_	^64^**Cu-ATSM T**_**max**_**/M**_**mean**_	^**64**^**Cu-ATSM T**_**mean**_**/M**_**mean**_	^**18**^**FDG SUV**_**max**_	^**18**^**FDG SUV**_**mean**_	**Max. pimonidazole labeled pixel value**	**Mean pimonidazole positive area fraction**
		Cm^3^								
1a	Fibrosarcoma	12.0	3.3	1.8	1.7	0.8	3.8	1.9	0.91	0.14
1b	After 45 Gy. RT	9.5	3.2	1.8	1.6	0.8	1.5	0.9	0.55	0.01
2	Squamous cell carcinoma	14.4	7.4	4.2	4.0	2.3	11.8	5.5	0.91	0.13
3	Undiff. sarcoma	12.3	6.8	4.0	4.1	2.0	9.1	5.6	0.94	0.10
4	Hemangiopericytoma	79.9	3.1	1.9	2.5	1.5	3.4	1.7	0.85	0.03
5	Fibrosarcoma	72.2	6.4	2.5	7.1	1.6	6.8	3.2	0.70	0.03
6	Fibrosarcoma	70.4	6.6	3.5	5.8	2.4	7.6	5.1	1.00	0.09
7	Hemangiopericytoma	13.8	1.2	0.6	N/A	N/A	2.7	1.3	0.55	0.03
8a	Fibrosarcoma	138.5	1.5	0.8	N/A	N/A	2.0	1.2	0.38	0.01
8b	After 45 Gy. RT	N/A	N/A	N/A	N/A	N/A	N/A	N/A	0.15	0.0

Tumor characteristics, PET imaging data and pimonidazole staining in canine tumors. For further details, please see text.

### Experimental setup

PET/CT scans were performed using a combined PET/CT scanner (Biograph 40, Siemens, Germany); consisting of a high-resolution PET-scanner and a 40-slice CT-scanner. CT parameters; slice thickness of 3.0 mm, 120 kV, 170 mAs, pitch 1.2, collimation 24 × 1.2 mm and a B30 kernel. The PET scans were acquired using a 3D acquisition mode and a 3D OSEM reconstruction (4 iterations, 8 subsets), and smoothed using a Gaussian filter having a FWHM of 3 mm, and a matrix size of 256 × 256. Tumor uptake of ^64^Cu-ATSM and ^18^FDG recorded on the PET/CT scans were determined using commercially available software (Pmod 3.0, Pmod Technologies, Switzerland) and reported in Table 1. In short, tumor volumes of interests (VOIs) were constructed manually and mean and maximum uptake values of ^64^Cu-ATSM and ^18^FDG determined. Muscle reference VOIs were placed manually in 5 slices on tumor adjacent musculature. All images and VOIs were evaluated by an experienced human PET physician and a board certified veterinary radiologist. ^18^FDG uptake levels were calculated and reported as standardized uptake values (SUV) and ^64^Cu-ATSM as tumor mean (T_mean)_ and tumor maximum (T_max_) to muscle mean (M_mean_) uptake ratios.

^18^FDG and ^64^Cu-ATSM PET/CT scans were performed before any biopsy procedures.^18^FDG PET/CT scans were performed on day 1 approximately 1 hour after the injection of ^18^FDG (injected activity 6.0-11.0 MBq/kg) and ^64^Cu-ATSM PET/CT scans were performed on the following two days approximately 3 and 24 hours after the injection of ^64^Cu-ATSM (injected activity 5.5 to 10.0 MBq/kg). Procedures performed on individual dogs are listed in table 1. Scanning and biopsy routines were as follows. Dogs received ^64^Cu-ATSM intravenously as a bolus. Ten to 30 min prior to this all dogs received an intravenous injection of 0.5 g Pimonidazole HCL/m^2^ body surface area suspended in 50 ml. of isotonic saline solution in accordance with a previously published procedure [17]. For scanning and biopsy procedures the dogs were pre-medicated with Methadone (0.2 to 0.3 mg/kg IM) and anesthetized using a bolus injection of Propofol. They were provided with 100 % oxygen via an endotracheal tube. Anesthesia was maintained by a continuous rate infusion of Propofol (15–25 mg/kg/hr). Heart rate, oxygen saturation, CO_2_ concentrations, and blood pressure were monitored throughout the procedures. Tumor biopsies were collected approximately 25 hours after the injection of Cu-ATSM in dogs 1 to 6. Biopsies from dog 7 where collected 10 hours after injection of Cu-ATSM. Dog 1 and 8 had biopsies collected both before, designated 1a and 8a, and after radiotherapy, designated 1b and 8b, respectively. Biopsies from dog 8 were collected 14 hours (8a) and 3 hours (8b) the injected of Cu-ATSM. Post radiotherapy biopsies were collected 6 weeks after completion of 45 Gy. radiation therapy (10 fractions of 4.5 Gy, three weekly treatments).

From each tumor biopsy set, three to nine individual biopsies, ranging from approximately 5 × 5 × 5 mm. to 10 × 10 × 10 mm. were collected from various regions within the tumor by a sterile procedure. Biopsies were snap-frozen in liquid nitrogen immediately after collection and subsequently several 10 μm sections were cut from each using a cryostat and then thaw mounted on poly-L-lysine coated microscopy slides.

### Autoradiography

The spatial distribution pattern of ^64^Cu-ATSM was determined by exposing tumor sections to phosphor imaging screens for 20 hours. These screens were read using a phosphor imaging system (Cyclone Plus Phosphor Imager, Perkin Elmer, Waltham, MA., USA), to form semi-quantitative photo-stimulated luminescence images of ^64^Cu-ATSM micro regional distributions. Tumor slides were subsequently frozen and stored at −80 °C until immunohistochemical analysis.

### Immunohistochemistry

Three to nine tumor sections from the individual tumors were evaluated microscopically for the distribution of bound pimonidazole. Immunohistochemical staining was performed using a previously described methodology [18]. Stained tumor sections were analyzed using a semiautomatic digital image acquisition system. Gray value images with a pixel size of 2.67 × 2.67 μm showing the distribution of pimonidazole were obtained and transformed at the pixel level into segmented binary images to visualize pimonidazole-positive cells using a previously published method [18].

### Image analysis

The spatial distribution patterns of ^64^Cu-ATSM photoluminescence autoradiography were compared to pimonidazole on the individual tumor sections using ImageJ (public domain, Java-based image processing program). All images were manually aligned and then cropped, based on masks drawn on pimonidazole immunofluorescence images to exclude areas of necrosis, dust particles and tissue holes. Images were subsequently rescaled to identical pixel sizes (100 × 100 μm), using pixel averaging without image interpolation. Regions of interest (ROIs) were manually drawn on pimonidazole immunofluorescence images, excluding the peripheral pixels to avoid influence of pixel averaging when downsizing.

Maximum pimonidazole labeled pixel value and mean pimonidazole positive labeled area fraction was calculated from the rescaled 100 × 100 μm pixel images. The pixel values on the rescaled on the 100 × 100 μm pixel images is the fraction of pimonidazole positive 2.67 × 2.67 μm pixels divided by the total number of 2.67 × 2.67 μm binary pixels composing the rescaled 100 × 100 μm pixels. Maximum pimonidazole labeled pixel value represent the single 100 × 100 μm pixel with the highest fraction of 2.67 × 2.67 μm pimonidazole positive pixels from all sections of the tumor. Mean pimonidazole positive labeled area fraction represent the average 100 × 100 μm image pixel value of all sections from the individual tumors.

Pimonidazole uptake characteristics of the individual tumors was examined and correlated to tumor PET uptake characteristics of ^64^Cu-ATSM and ^18^FDG from the previously performed PET scans. Mean pimonidazole positive area fraction and maximum pimonidazole pixel values were correlated to maximum and mean tumor to muscle uptake ratios of ^64^Cu-ATSM and maximum and mean SUV of ^18^FDG.

**Figure 1:**
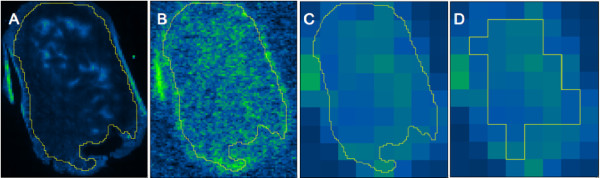
**Pixel-by-pixel correlation.** Methodology for pixel-by-pixel correlation analysis of pimonidazole immunostainings and ^64^Cu-ATSM autoradiography images. **A**, 100 × 100 μm pimonidazole image, with original ROI excluding folding and artifacts. **B**, 100 × 100 μm ^64^Cu-ATSM autoradiography image, identical ROI as image **A**. **C**, ^64^Cu-ATSM image rescaled from a pixel size of 100 × 100 μm to 1×1 mm, identical ROI as image **A**. **D**, new ROI applied with only pixels not transversed by the original ROI from image **A** included. Soft tissue sarcoma, displaying a weak insignificant correlation (r_s_ = 0.30, p-value: 0.12) between the two modalities.

The 100 × 100 μm pimonidazole immunofluorescence and corresponding co-registered 100 × 100 μm ^64^Cu-ATSM autoradiography images were rescaled to a pixel size of 1 × 1 mm by pixel averaging without interpolation. The two-step rescaling procedure allowed for the recognition and exclusion of artifacts within both immunohistochemical and autoradiography images at similar resolution. The manual ROIs from original images were applied to the 1 × 1 mm pixel size images and only pixels not transversed by the section-delineating ROI were included during subsequent pixel-by-pixel correlation analysis (Figure 1).

### Statistical analysis

Statistical analyses were performed using Graph Pad Prism (Version 5.0b, GraphPad, Inc. La Jolla, Ca, USA). All correlations were performed as non-parametric Spearman’s rank correlation analysis. For all statistical analysis a p-value < 0.05 was considered significant.

## Results

Maximum pimonidazole labeled pixel value ranged from 1.0 to 0.15, while pimonidazole positive area fraction ranged from 0.14 to 0.0 between tumors.

The ^64^Cu-ATSM T_max_/M_mean_ uptake ratio deduced from images acquired 3 hours pi. was found to be positively correlated to maximum pixel value in pimonidazole labeled images with a correlation coefficient of 0.81 *(p = 0.011)*, and to pimonidazole positive fractions by a correlation coefficient of 0.69 *(p = 0.043)*. The ^64^Cu-ATSM T_mean_/M_mean_ uptake ratio at 3 hours pi. was also significantly correlated to maximum pimonidazole pixel values, by a correlation coefficient of 0.77 *(p = 0.021)*. Mean and maximum ^18^FDG SUV displayed a positive correlation to pimonidazole staining values. Maximum ^18^FDG SUV was correlated to both pimonidazole maximum and pimonidazole positive area fraction values with correlation coefficients of 0.82 *(p = 0.008)* and 0.79 *(p = 0.014)*, respectively. Mean ^18^FDG SUV correlated to maximum and mean pimonidazole pixel values with correlation coefficients of 0.85 *(p = 0.008)* and 0.77 *(p = 0.017)*, respectively. Correlation between ^64^Cu-ATSM T_mean_/M_mean_ 3 hours pi. and mean pimonidazole positive area fraction and all correlations between ^64^Cu-ATSM uptake 24 hours pi. and pimonidazole and were found to be statistically insignificant (Table 2).

**Table 2:** **FDG and**^**64**^**Cu-ATSM PET uptake vs. Pimonidazole staining**

	Maximum pimonidazole labeled pixel value	Mean pimonidazole positive area fraction
^64^Cu-ATSM T_max_/M_mean_ 3 h.	**0.81 (p = 0.011)**	**0.69 (p = 0.043)**
^64^Cu-ATSM T_mean_/M_mean_ 3 h.	**0.77 (p = 0.021)**	0.56 (p = 0.126)
^64^Cu-ATSM T_max_/M_mean_ 24 h.	0.40 (p = 0.396)	0.036 (p = 0.964)
^64^Cu-ATSM T_mean_/M_mean_ 24 h.	0.70 (p = 0.088)	0.24 (p = 0.595)
^18^FDG SUV_max_	**0.82 (p = 0.008)**	**0.79 (p = 0.014)**
^18^FDG SUV_mean_	**0.85 (p = 0.008)**	**0.77 (p = 0.017)**

Pimonidazole maximum labeled pixel value and mean pimonidazole positive area fraction of individual tumors correlated to their ^64^Cu-ATSM maximum and mean T/M ratios 3 and 24 h. pi. and ^18^FDG maximum and mean SUV. Spearman non-parametric correlation coefficients and *p-value*. Significant correlations in bold.

**Figure 2:**
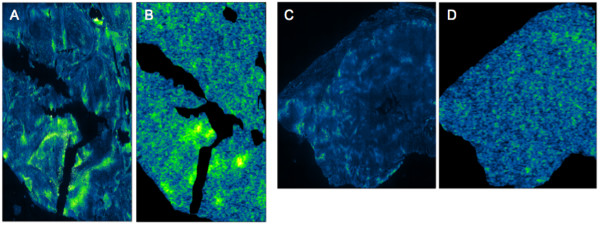
**Pimonidazole immunofluorescence and**^**64**^**Cu-ATSM autoradiography.** Pimonidazole immunofluorescence (**A** and **C**) and ^64^Cu-ATSM photoluminescence autoradiography (**B** and **D**) of tumor sections from a fibrosarcoma (**A** and **B**) and an undifferentiated sarcoma (**C** and **D**). The fibrosarcoma displayed high and heterogeneous pimonidazole immunofluorescence staining (A) whereas lower and more homogeneous pimonidazole staining was found in the undifferentiated sarcoma (**D**). A strong correlation (r_s_ = 0.72; p < 0.001) with ^64^Cu-ATSM was found in the fibrosarcoma (**A** vs. **B**) whereas this was not the case in the undifferentiated sarcoma (**C** vs. **D**)

Pixel-by-pixel correlation analysis of ^64^Cu-ATSM autoradiography and pimonidazole immunofluorescence yielded varying results with correlation coefficients of individual sections ranging from a strong positive correlation of 0.88 to a moderate negative of −0.56). The correlation coefficients were only statistically significant in 15 out of 55 sections. Twelve sections displayed a statistically significant positive correlation, ranging from 0.41 to 0.88 and three sections from one tumor, displayed a significant negative correlation ranging from −0.35 to −0.56. Figure 2 illustrates two sections with positive and no correlation, respectively. The sections from the tumor of dog no.6 displayed the most statistically significant positive correlation, with significant correlation coefficients ranging from 0.41 to 0.77 (mean 0.63) in six of nine evaluated sections.

## Discussion

The observed correlations between ^64^Cu-ATSM PET uptake and pimonidazole staining characteristics indicate that high ^64^Cu-ATSM uptake on PET, at least at 3 hours pi., is indicative of intratumoral hypoxia as measured by pimonidazole immunostainings. The validity of comparing Cu-ATSM PET uptake at 3 hours pi and pimonidazole immunohistochemistry on biopsies collected up to 25 hours after administration can be discussed. Previous studies on pimonidazole in canine tumors have reported that adduct formation occurs from within 20 minutes and sufficient antigen persists for days to allow for acceptable immunohistochemistry [17]. This together with the pimonidazole adduct formation being irreversible in hypoxic cells and its relatively short plasma half-life [15,17], makes a comparison of 3 hour Cu-ATSM PET and pimonidazole valid. The lack of information on the reversibility of cellular Cu-ATSM binding justifies similar considerations for the comparison of Cu-ATSM PET 24 hour pi. uptake characteristics and pimonidazole.

The maximum and mean SUV of ^18^FDG on PET images displayed a strong positive correlation to pimonidazole staining characteristics. Interestingly, based on these correlations, ^64^Cu-ATSM tumor PET uptake appears not to improve on ^18^FDG uptake as a hypoxia marker where hypoxia is assessed by pimonidazole immunohistochemistry on tumor biopsies. However, no information on the spatial correlation between ^18^FDG and pimonidazole was available.

The correlation between pimonidazole and uptake of the two markers ^18^FDG and ^64^Cu-ATSM could indicate that hypoxic tumors display increased glycolytic activity (Pasteur effect) [19] or that highly glycolytic tumors might have a more aggressive growth potential, which increases the risk of forming hypoxic tumor regions [20]. There are limitations in this interpretation given the low number of tumors, the different histopathologies, and the potential for different baseline glycolytic activity irrespective of hypoxia. Previous studies assessing the potential of ^18^FDG as a surrogate marker of hypoxia have reported similar concerns. [21].

The wide spectrum of correlations between ^64^Cu-ATSM autoradiography and pimonidazole immunofluorescence is indicated by Spearman’s rank correlation coefficients ranging from strong positive to moderate negative from the individual tumor sections. A clear tendency for increased positive correlation was observed in tumors and sections displaying higher maximum and mean pimonidazole labeling fractions. A negative correlation between the two modalities was only observed for one tumor, which suggests that potential tumor dependent differences could exist.

The distribution of the two tracers in tumor sections displaying a heterogeneous uptake patterns indicate that ^64^Cu-ATSM, at least in these sections, is an indicator of regional tumor hypoxia as assessed by pimonidazole. No firm conclusions into to the can be made on the data reported here. Based on the different uptake mechanisms of Cu-ATSM and pimonidazole, including differences in oxygen levels required for induction and maximal binding, a completely similar microregional distribution can probably not be expected from the two labels. The pimonidazole positive staining region on the binary segmented images ranged from 0 to 0.14 between tumors, which means that in the major part of sections, the few scattered pimonidazole positive pixels seen were likely the result of random noise and would not be expected to have corresponding ^64^Cu-ATSM activity. In contrast, regions displaying more intense activity, some correlation was seen. In these higher uptake areas correlation coefficients indicate that ^64^Cu-ATSM accumulates in patterns that to some extent are comparable to those of pimonidazole. However, despite this trend, one tumor displayed a negative correlation between the two tracers, which could be comparable to previous observation in preclinical studies [10,11]. Cycling changes in hypoxia distribution have been identified as a hallmark feature of solid tumors [22]. Since pimonidazole adduct formation is irreversible and if for the sake of argument Cu-ATSM accumulation is reversible in cells experiencing re-oxygenation then varying correlations observed between Cu-ATSM 24 hours pi. and pimonidazole can be explained. Pixel-by-pixel comparisons were performed at a pixel size of 1 × 1 mm, which limits the possibility to detect minor spatial differences between the two tracers. However, the use of this pixel size decreases both image noise and the influence of minor inaccuracies in the co-registration process. It could be argued that a larger pixel size would add additional information and better mimic the resolution of modern PET scanners [23], however increasing pixel sizes beyond 1 × 1 mm was not reasonable considering the sizes of tumor sections used. Biopsies were for most tumors obtained after approximately 25 hours. The optimal period allowed for the distribution of ^64^Cu-ATSM in spontaneous canine tumors is not reported. It is possible that no general optimal period exists as based on previously reported findings in xenografted models, the distribution period necessary for ^64^Cu-ATSM to attain sufficient hypoxia-specificity varies widely among tumors [11].

The tumor sections from biopsies obtained after shorter distribution periods than approximately 25 hours displayed no improved pimonidazole staining or ^64^Cu-ATSM uptake or heterogeneity. These sections did however only display limited accumulation of both tracers and more intensively staining sections could potentially show a benefit with regard to earlier sampling time. We have previously reported a delayed accumulation of Cu-ATSM in hypoperfused tumor regions [16]. The tumor of dog no. 6 was included in the previous study and displayed a central hypoperfused tumor core with delayed accumulation of Cu-ATSM. Interestingly, six of nine sections from this tumor displayed a statistically significant moderate to strong positive correlation between Cu-ATSM and pimonidazole after a distribution period of 25 hours, indicating that, at least in this tumor, prolonged distribution periods for Cu-ATSM may be required.

There are several limitations to this study, in particular the low number of tumors investigated, their varying histopathologies and the need for general anesthesia during procedures. The validity of comparing universal tumor uptake levels of ^18^FDG and ^64^Cu-ATSM, assessed by PET imaging, to regional uptake levels on segmented pimonidazole immunostainings is also questionable. Furthermore, the microregional spatial comparison of two different hypoxia markers with different uptake mechanisms and probably differences in oxygen level dependence for accumulation, cannot be expected to be exactly identical and directly comparable. Non-invasive and invasive imaging studies of tumor hypoxia may additionally be affected by long periods between injection of tracers and image acquisition considering the varying survival time and turn over of hypoxic tumor cells reported [14].

## Conclusions

In conclusion this study provides several indications that ^64^Cu-ATSM accumulation patterns are comparable to those of pimonidazole in several spontaneous tumors available in this study. It also suggests that the hypoxia specificity of ^64^Cu-ATSM may differ between tumors and with level of hypoxia.

Validation against invasive measures of hypoxia is important in the evaluation and development of non-invasive alternatives. In this perspective spontaneous clinical animal models hold the potential to bridge the numerous gaps between pre-clinical induced cancer models and human cancer patients.

## Competing interests

The authors declare that they have no competing interests.

## Author contributions

AEH carried out the PET/CT scans, collected biopsies, performed autoradiograhy, image analysis and statistics, planned study and drafted manuscript. ATK participated in the planning of the study and helped to draft the manuscript. JTJ performed autoradiography and image analysis. FJM performed image analysis, statistic and helped draft the manuscript. MB assisted in planning the study and image analysis. AJK performed pimonidazole immunofluorescence and segmentation of images. JB performed pimonidazole immunofluorescence and segmentation of images. SAE participated in the planning of the study and helped to draft the manuscript. AK participated in the planning of the study, autoradiography and image analysis and helped to draft the manuscript. All authors read and approved the final manuscript.
